# Personal

**Published:** 1892-12

**Authors:** 


### Personal.

DR. HORACE CLARK announces that he limits his practice to diseases of the nose, throat, and chest. Consultation hours, 9 to 2. No. 21 West North street, Buffalo. Telephone, 1650.

